# Long non-coding RNA CASC2 enhanced cisplatin-induced viability inhibition of non-small cell lung cancer cells by regulating the PTEN/PI3K/Akt pathway through down-regulation of miR-18a and miR-21

**DOI:** 10.1039/c8ra00549d

**Published:** 2018-04-30

**Authors:** Li Li, Haifeng Zhang, Xiaolong Wang, Jiali Wang, Haitao Wei

**Affiliations:** School of Nursing and Health, Henan University Kaifeng 475004 P. R. China; Department of Thoracic Surgery, Huaihe Hospital of Henan University Baobei Road No. 8 Kaifeng 475000 P. R. China sea_wei88@sina.com +86-037123906663; Department of Surgery, Huaihe Hospital of Henan University Kaifeng 475000 P. R. China

## Abstract

Long non-coding RNA cancer susceptibility candidate 2 (lncRNA CASC2) is a tumor suppressor and has been proved to contribute to chemotherapy efficacy. However, the effect of CASC2 on cisplatin cytotoxicity in non-small cell lung cancer (NSCLC) is unclear. The present study aimed to investigate the role of CASC2 in regulating cisplatin cytotoxicity in NSCLC cells. Herein, we found that CASC2 was low-expressed, while miR-18a and miR-21 were over-expressed in NSCLC cell lines. CASC2 enhanced the inhibition effect of cisplatin on cell viability. Down-regulation of miR-18a and miR-21 exhibited the similar effect to CASC2 and mimics of miR-18a and miR-21 displayed the opposite effect to CASC2. MiR-18a and miR-21 were two targets of CASC2 in NSCLC. PTEN was found to be a target of miR-18a and miR-21 in NSCLC. CASC2 overexpression increased PTEN expression level and reduced the ratio of p-Akt/Akt. MiR-18a or miR-21 mimics attenuated the effect of CASC2 overexpression on the PTEN expression and ratio of p-Akt/Akt. The results suggested that CASC2 enhanced cisplatin-induced viability inhibition of NSCLC cells *via* PTEN/PI3K/Akt pathway through suppressing miR-18a and miR-21 expression.

## Abstract 2

Long non-coding RNA cancer susceptibility candidate 2 (lncRNA CASC2) is a tumor suppressor and has been proved to contribute to chemotherapy efficacy.

## Graphical abstract

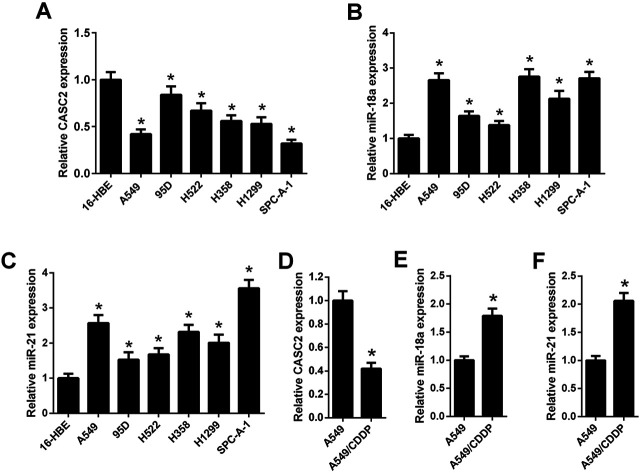

## Introduction

Lung cancer is one of the most common cancers and the leading cause of cancer-related mortality worldwide.^1^ There are two main types of lung cancer: small cell lung cancer (SCLC) and non-small cell lung cancer (NSCLC).^2^ It has been reported that NSCLC accounts for approximately 85% of all cases of lung cancer.^2^ For early stages of NSCLC, the treatment is surgery, but for some locally advanced cancers and metastatic disease, the treatments are chemotherapy with concurrent radiation and palliative chemotherapy, respectively.^2^ Cisplatin is an effective chemotherapeutic drug, which is usually used for the treatment of many cancers, however, the effect of cisplatin is diminished because of the drug resistance.^3^ Cisplatin resistance has been found in the treatment of NSCLC.^4^

Long non-coding RNAs (lncRNAs) are a group of non-protein-coding RNAs.^5^ Recently, aberrant lncRNAs expression is observed in many kinds of cancers, and lncRNAs have caught increasing attention for their roles in cancer progression.^6^ Cancer susceptibility candidate 2 (CASC2) is a member of lncRNAs and is known as a novel tumor suppressor.^7^ CASC2 has been reported to modulate resistance to temozolomide (TMZ)-based chemotherapy in glioma tumorigenesis.^8,9^ However, the effect of CASC2 on cisplatin cytotoxicity in non-small cell lung cancer (NSCLC) is unknown.

MicroRNAs (miRNAs) are another group of non-coding RNAs, which are important posttranscriptional regulators.^10,11^ MiRNAs play crucial roles in various biological processes, and aberrant miRNAs expression is associated with many kinds of diseases, including cancers.^12^ For instance, miR-18a and miR-21 are found to be over-expressed in NSCLC.^13,14^ It is well known that lncRNAs may interact with miRNAs and regulate the expression of their target miRNAs.^15^ Moreover, miR-18a and miR-21 have been demonstrated to be the targets of CASC2.^16,17^

In the present study, the role of CASC2 in regulating cisplatin cytotoxicity in NSCLC cells was investigated. We found that CASC2 enhanced cisplatin-induced viability inhibition of NSCLC cells by regulating the PTEN/PI3K/Akt pathway through down-regulation of miR-18a and miR-21.

## Materials and methods

### Cell culture

The human bronchial epithelial cell line (16-HBE) and six NSCLC cell lines (A549, 95D, H522, H358, H1299, and SPC-A-1) were obtained from the Cell Bank of Type Culture Collection of the Chinese Academy of Sciences (Shanghai, China). The cisplatin-resistant A549 cell line (A549/CDDP) was purchased from Biosis Biotechnology Co., Ltd. (Shanghai, China). Cells were cultured in Dulbecco's modified Eagle's medium (Invitrogen, Carlsbad, CA, USA) supplemented with 10% fetal bovine serum (Gibco, Carlsbad, CA, USA), 100 U ml^−1^ penicillin, and 100 μg ml^−1^ streptomycin. Cells were cultured in a humidified atmosphere of 5% CO_2_ at 37 °C.

### qRT-PCR

Total RNA was extracted from cells using Trizol reagent (Invitrogen). The total RNA were reverse transcribed with a Transcriptor First Strand cDNA Synthesis Kit (Takara, Dalian, China). The SYBR PremeScript miRNA RT-PCR kit (Takara) was used for qRT-PCR according to the manufacturer's instruction. The expression of mRNA and miRNA was normalized to GAPDH (for CASC2) or U6 (for miR-18a and miR-21) expression level, respectively. The primers were as follows: CASC2, forward 5′-GCAC ATTG GACG GTGT TTCC-3′, reverse 5′-CCCA GTCC TTCA CAGG TCAC-3′; miR-18a: forward 5′-TCCG AGAT AGAC GTGA TCTA-3′, reverse 5′-GTGC AGGG TCCG AGGT-3′; miR-21: forward 5′-GGGT AGCT TATC AGAC TGAT GTT-3′, reverse 5′-CAGT GCAG GGTC CGAG GT-3′; U6, forward 5′-CTCG CTTC GGCA GCAC ATAT ACT-3′, reverse 5′-ACGC TTCA CGAA TTTG CGTG TC-3′; GAPDH, forward 5′-AATG GGCA GCCG TTAG GAAA-3′, reverse 5′-TGAA GGGG TCAT TGAT GGCA-3′. The 2^−ΔΔ*C*_t_^ method was used to quantify the expression fold changes.

### Transfection

The transfection was performed using Lipofectamine 2000 (Invitrogen) according to the instructions of the manufacturer. The transfected cells were cultured for 48 h before analysis. The CASC2 overexpression vector (pcDNA3.1-CASC2), empty vector (pcDNA3.1), miR-18a inhibitor, miR-21 inhibitor, inhibitor control, miR-18a mimics, miR-21 mimics and mimic control were synthesized by Shanghai GenePharma Co., Ltd. (Shanghai, China).

### MTT assay

To evaluate the cell viability, MTT assay was performed. Briefly, A549, H1299, and SPC-A-1 cells with or without transfection were seeded in 96-well plates at the density of 5 × 10^3^ cells per well and incubated with different concentrations of cisplatin (0, 2.5, 5, 7.5, 10, and 12.5 μM). For A549/CDDP cells, the concentrations of cisplatin were 0, 2, 4, 8, 16, 32 and 64 μM. After 48 h of incubation, 10 μl of MTT solution (5 mg ml^−1^) was added into each well and incubated for 4 h. Then the DMSO solution was added to dissolve the formed formazan. Finally, the absorbance at 490 nm was measured with a microplate reader (Bio-Rad Laboratories, Hercules, CA, USA).

### Luciferase reporter gene assays

The fragments of the 3′UTR of CASC2 and PTEN were amplified and cloned into the pMiR-Report reporter plasmid (Ambion Inc., Austin, TX, USA), respectively. The mutated 3′UTR of CASC2 or PTEN was produced by two-step PCR approach and also cloned in to the pMiR-Report. To evaluate the interaction of CASC2 and miR-18a, the reporter vectors containing the wide type CASC2 (CASC2-WT) or mutated CASC2 (CASC2-MUT) and miR-18a mimics or mimic control were co-transfected into A549 cells. To evaluate the interaction of CASC2 and miR-21, the reporter vectors containing CASC2-WT or CASC2-MUT and miR-21 mimics or mimic control were co-transfected into A549 cells. To assess whether PTEN is a target of miR-18a, the reporter vectors containing the wide type PTEN (PTEN-WT) or mutated PTEN (PTEN-MUT) and miR-18a mimics or mimic control were co-transfected into A549 cells. To confirm whether PTEN is a target of miR-21, the reporter vectors containing PTEN-WT or PTEN-MUT and miR-21mimics or mimic control were co-transfected into A549 cells. After 48 h, the luciferase activity was measured using the Dual-Luciferase reporter assay system (Promega, Madison, WI, USA). The Renilla luciferase activity was normalized to the firefly luciferase activity.

### Western blotting

Total proteins were extracted from cells using protein extraction reagent RIPA (Beyotime Biotechnology, Shanghai, China). The protein concentration was determined using a protein assay kit (Bio-Rad). Then the protein lysates (50 μg) were separated on 12% SDS-PAGE and blotted onto nitrocellulose membranes (Bio-Rad). Membranes were blocked with 5% non-fat milk in Tris buffered saline for 2 h at room temperature. Subsequently, the membrane was incubated with specific primary antibodies against PTEN, Akt, p-Akt (S473), and β-actin (Abcam, Cambridge, MA, USA) overnight at 4 °C. The membrane was then incubated with horseradish peroxidase (HRP)-conjugated secondary antibody (Abcam) at room temperature for 2 h. Finally, the immune-reactivity was visualized using the enhanced chemiluminescence (ECL) reagent (Bio-Rad) according to the manufacturer's protocols.

### Statistical analysis

All data are presented as mean ± SD. Statistical significance was determined with Student's *t* test or one-way ANOVA using SPSS version 22.0 software (IBM, Chicago, IL, USA). *p* < 0.05 was considered statistically significant.

## Results

### The expression levels of CASC2, miR-18a, and miR-21 in NSCLC cells

**Fig. 1:**
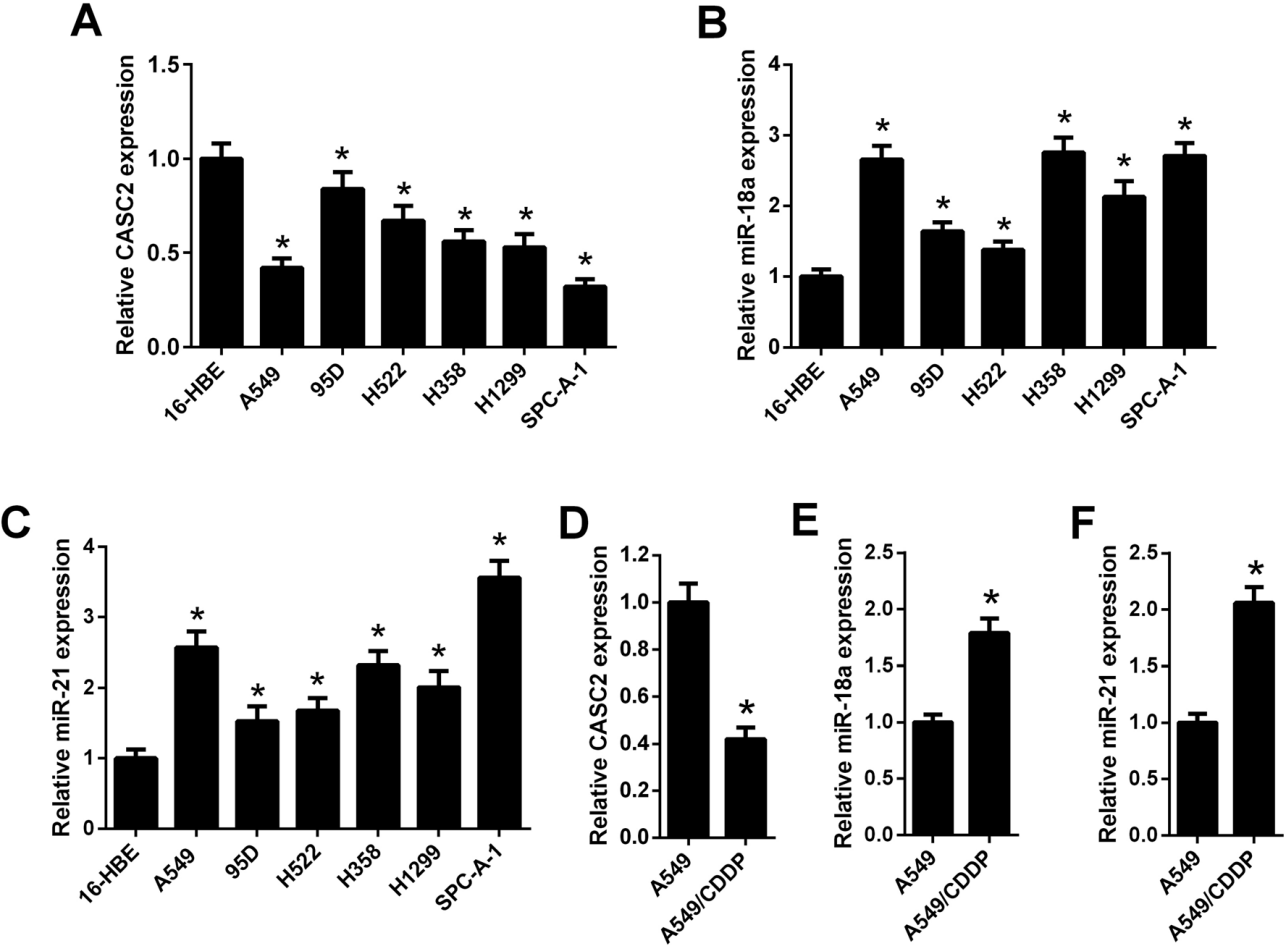
The expression levels of CASC2, miR-18a, and miR-21 in NSCLC cells. (A) The relative expression levels of CASC2 were measured using qRT-PCR in the human bronchial epithelial cell line (16-HBE) and six NSCLC cell lines (A549, 95D, H522, H358, H1299, and SPC-A-1). *p < 0.05 *vs.* 16-HEB cells, *n* = 3. (B and C) The relative expression levels of miR-18a and miR-21were measured using qRT-PCR. *p < 0.05 *vs.* 16-HEB cells, *n* = 3. (D–F) The relative expression levels of CASC2, miR-18a, and miR-21 in A549 and cisplatin-resistant A549 (A549/CDDP) cells. *p < 0.05 *vs.* A549 cells, *n* = 3.

The relative expression levels of CASC2 were measured using qRT-PCR in NSCLC cells. The results in Fig. 1A showed that CASC2 was low-expressed in NSCLC cells, including A549, 95D, H522, H358, H1299, and SPC-A-1, compared with the 16-HEB cells. Besides, the expression levels of miR-18a and miR-21 in NSCLC cells were also detected by qRT-PCR. The results in Fig. 1B and C showed that miR-18a and miR-21 were high-expressed in NSCLC cells. A cisplatin-resistant A549 cell line (A549/CDDP) was used to observe whether the expression levels of CASC2, miR-18a, and miR-21 were changed between cisplatin-sensitive and cisplatin-resistant NSCLC cells. As shown in Fig. 1D, CASC2 level was decreased in A549/CDDP cells compared with A549 cells. The levels of miR-18a and miR-21 were increased in A549/CDDP cells compared with A549 cells (Fig. 1E and F).

### Evaluation of cisplatin cytotoxicity to NSCLC cells

**Fig. 2:**
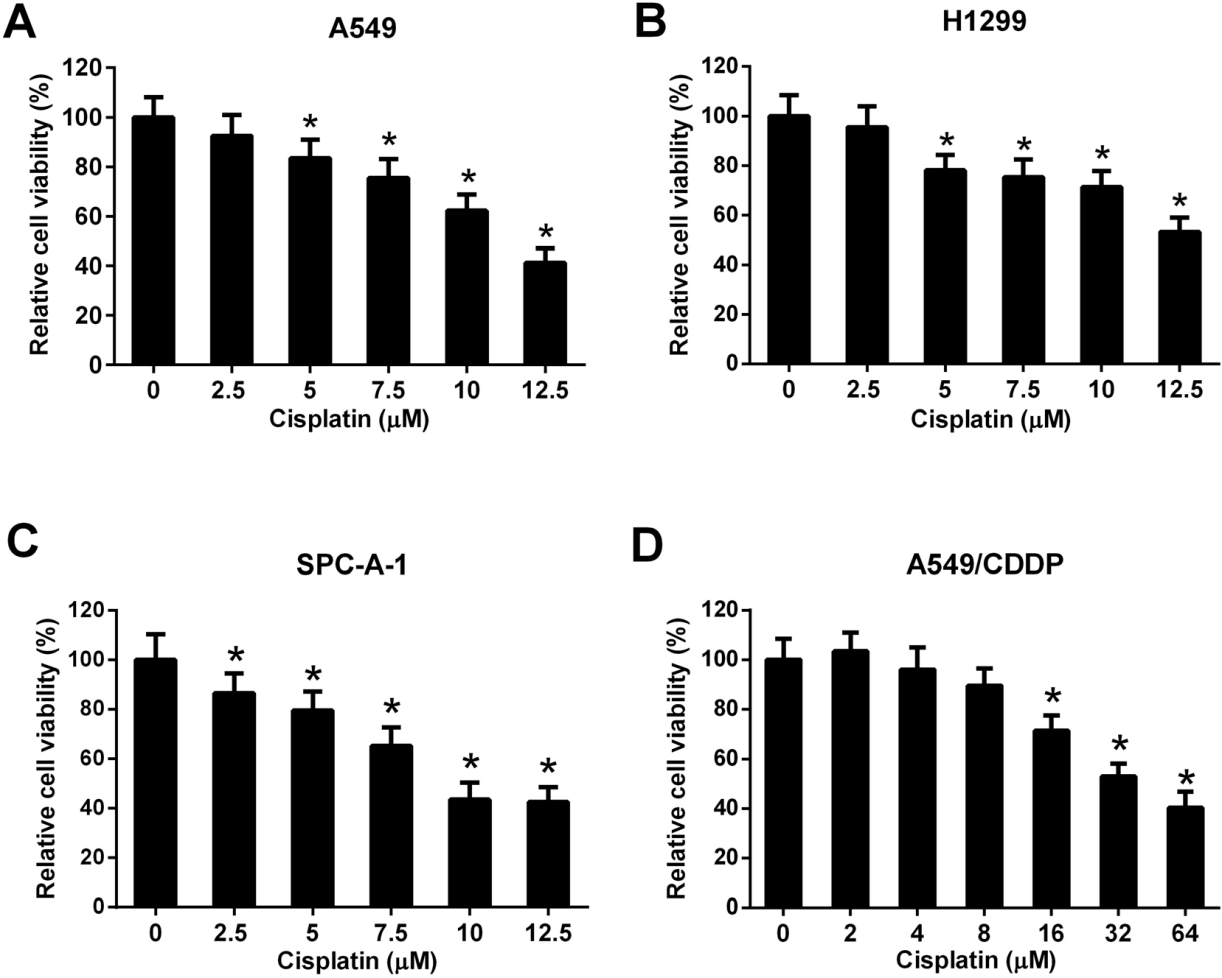
Evaluation of cisplatin cytotoxicity to NSCLC cells. A549, H1299, and SPC-A-1 cells were incubated with 0, 2.5, 5, 7.5, 10, and 12.5 μM of cisplatin for 48 h. A549/CDDP cells were incubated with 0, 2, 4, 8, 16, 32, and 64 μM of cisplatin for 48 h. The cell viability was detected by MTT assay. (A) The viability of A549 cells. *p < 0.05 *vs.* A549 cells without cisplatin treatment, *n* = 3. (B) The viability of H1299 cells. *p < 0.05 *vs.* H1299 cells without cisplatin treatment, *n* = 3. (C) The viability of SPC-A-1 cells. *p < 0.05 *vs.* SPC-A-1 cells without cisplatin treatment, *n* = 3. (D) The viability of A549/CDDP cells. *p < 0.05 *vs.* A549/CDDP cells without cisplatin treatment, *n* = 3.

Three NSCLC cell lines (A549, H1299, and SPC-A-1) were selected to evaluate the cytotoxicity effect of cisplatin on NSCLC cells. Cells were incubated with different concentrations of cisplatin (0, 2.5, 5, 7.5, 10, and 12.5 μM) for 48 h and the cell viability was detected by MTT assay. The results showed that cisplatin with the concentration of 5, 7.5, 10, and 12.5 μM significantly inhibited the cell viability of A549 and H1299 cells (Fig. 2A and B). Cisplatin with the concentration of 2.5, 5, 7.5, 10, and 12.5 μM significantly inhibited the cell viability of SPC-A-1 cells (Fig. 2C). A549/CDDP cells were incubated with different concentrations of cisplatin (0, 2, 4, 8, 16, 32, and 64 μM) for 48 h. The results of MTT assay showed that cisplatin with the concentration of 16, 32, and 64 μM inhibited the viability of A549/CDDP cells (Fig. 2D).

### Effect of CASC2, miR-18a, and miR-21 on cisplatin-induced viability inhibition of NSCLC cells

**Fig. 3:**
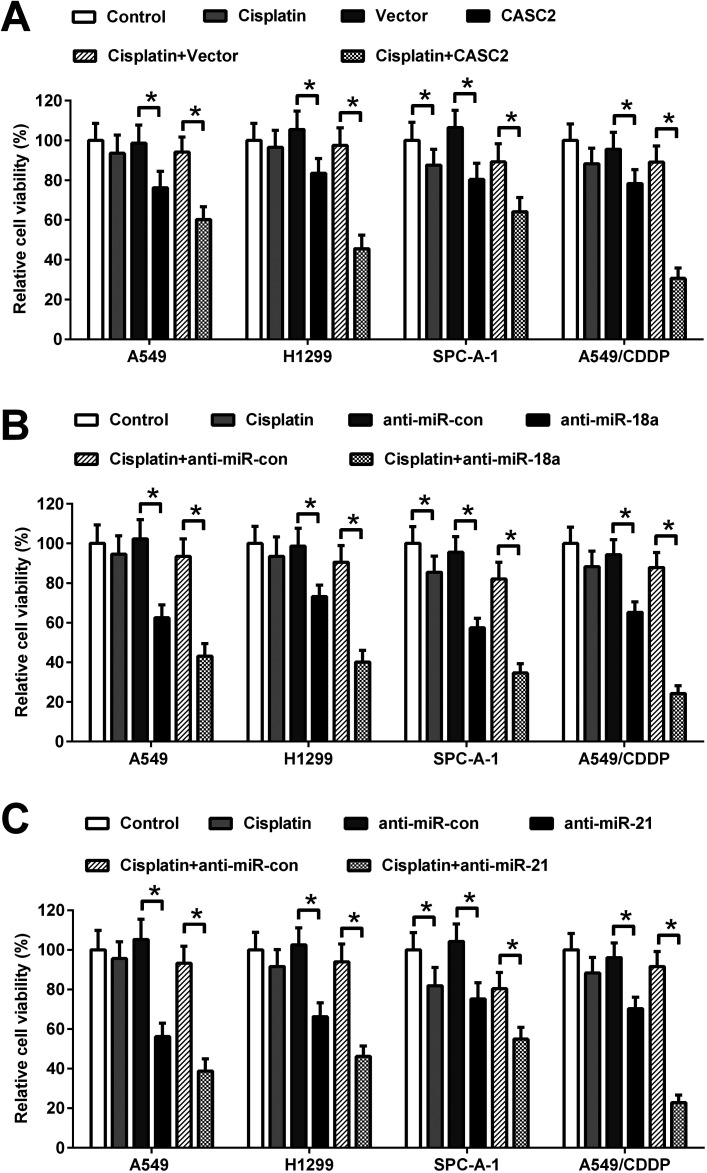
Effect of CASC2, miR-18a, and miR-21 on cisplatin-induced viability inhibition of NSCLC cells. (A) Cells were transfected with the CASC2 overexpression vector (pcDNA3.1-CASC2) or empty vector (pcDNA3.1) and incubated with or without cisplatin (2.5 μM for A549, H1299, and SPC-A-1 cells; 8 μM for A549/CDDP cells). The viability of A549, H1299, SPC-A-1, and A549/CDDP cells was detected by MTT assay. (B) Cells were transfected with miR-18a inhibitor or inhibitor control, and incubated with or without cisplatin (2.5 μM for A549, H1299, and SPC-A-1 cells; 8 μM for A549/CDDP cells). The cell viability was detected by MTT assay. (C) Cells were transfected with miR-21 inhibitor or inhibitor control, and incubated with or without cisplatin (2.5 μM for A549, H1299, and SPC-A-1 cells; 8 μM for A549/CDDP cells). The cell viability was detected by MTT assay. *p < 0.05, *n* = 4.

To investigate the effect of CASC2, miR-18a, and miR-21 on cisplatin-induced viability inhibition, the CASC2 overexpression vector (pcDNA3.1-CASC2), empty vector (pcDNA3.1), miR-18a inhibitor, miR-21 inhibitor, or inhibitor control was transfected into A549, H1299, SPC-A-1, and A549/CDDP cells. Subsequently, A549, H1299, and SPC-A-1 cells were incubated with or without 2.5 μM of cisplatin and A549/CDDP cells were incubated with or without 8 μM of cisplatin for 48 h. Cell viability was detected by MTT assay. As shown in Fig. 3A, CASC2 overexpression inhibited viability of A549 cells, H1299, SPC-A-1, and A549/CDDP cells. CASC2 overexpression enhanced the inhibitory effect of cisplatin. MiR-18a and miR-21 inhibitors also exhibited inhibitory effect on viability of A549, H1299, SPC-A-1, and A549/CDDP cells. Furthermore, miR-18a and miR-21 inhibitors enhanced cisplatin-induced viability inhibition of A549, H1299, SPC-A-1, and A549/CDDP cells (Fig. 3B and C).

### MiR-18a and miR-21 were direct targets of CASC2 in NSCLC cells

**Fig. 4:**
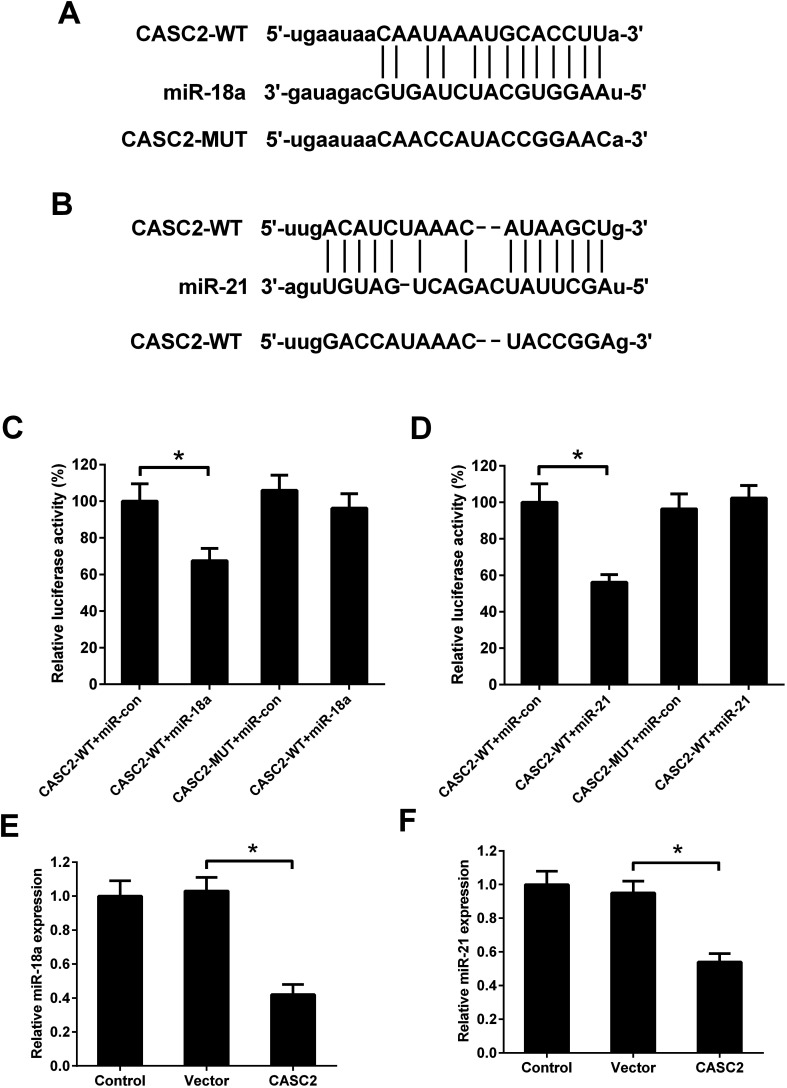
MiR-18a and miR-21 were direct targets of CASC2. (A) Predicted binding sites between CASC2 and miR-18a. (B) Predicted binding sites between CASC2 and miR-21. (C and D) Luciferase reporter assay was performed in A549 cells. (E and F) The expression levels of miR-18a/miR-21 in A549 cells transfected with CASC2 overexpression vector (pcDNA3.1-CASC2) or empty vector (pcDNA3.1). *p < 0.05, *n* = 3.

Although the interaction between CASC2 and miR-18a/miR-21 has been predicted by computational algorithms (Fig. 4A and B) and confirmed by previous experiments,^16,17^ it is unknown whether CASC2 could regulate expression of miR-18a and miR-21 in NSCLC cells. Luciferase reporter assay was performed to determine the binding of CASC2 and miR-18a/miR-21. The results in Fig. 4C showed that the luciferase activity of A549 cells transfected with CASC2-WT and miR-18a mimics was decreased compared with the cells transfected with CASC2-WT and mimic control. The results in Fig. 4D showed that co-transfection with CASC2-WT and miR-21 mimics inhibited the luciferase activity of A549 cells, compared to co-transfection with CASC2-WT and mimic control. The results in Fig. 4E and F suggested that CASC2 overexpression suppressed the expression levels of miR-18a and miR-21. All data indicated that miR-18a and miR-21 were direct targets of CASC2 in A549 cells.

### MiR-18a and miR-21 resisted the effect of CASC2 on viability of NSCLC cells

**Fig. 5:**
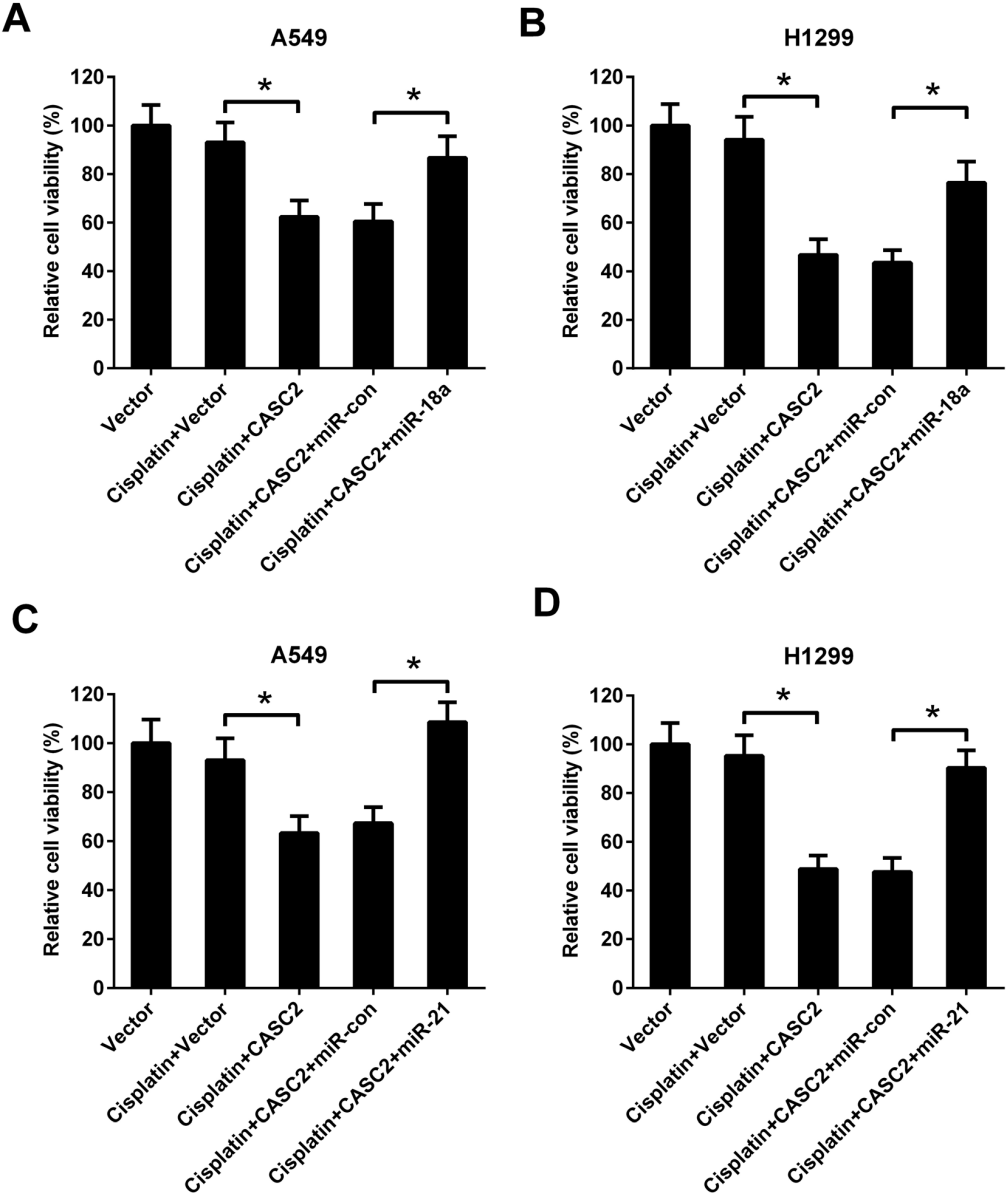
MiR-18a and miR-21 resisted the effect of CASC2 on viability of NSCLC cells. To further investigate the role of miR-18a and miR-21 in the effect of CASC2 on NSCLC cell viability, A549 (A and C) and H1299 cells (B and D) were co-transfected with pcDNA3.1-CASC2 and miR-18a/miR-21 mimics. The cells were incubated with cisplatin (2.5 μM) for 48 h. The cell viability was detected by MTT assay. *p < 0.05, *n* = 4.

To further investigate the role of miR-18a and miR-21 in the effect of CASC2 on NSCLC cell viability, A549 and H1299 cells were co-transfected with pcDNA3.1-CASC2 and miR-18a/miR-21 mimics. As shown in Fig. 5A and C, CASC2 overexpression enhanced the inhibition effect of cisplatin on cell viability, while miR-18a or miR-21 up-regulation significantly reversed the effect of CASC2 overexpression in A549 cells. Moreover, miR-18a or miR-21 up-regulation also attenuated the effect of CASC2 overexpression on cisplatin-induced viability inhibition in H1299 cells (Fig. 5B and D).

### PTEN was a direct target of miR-18a and miR-21

**Fig. 6:**
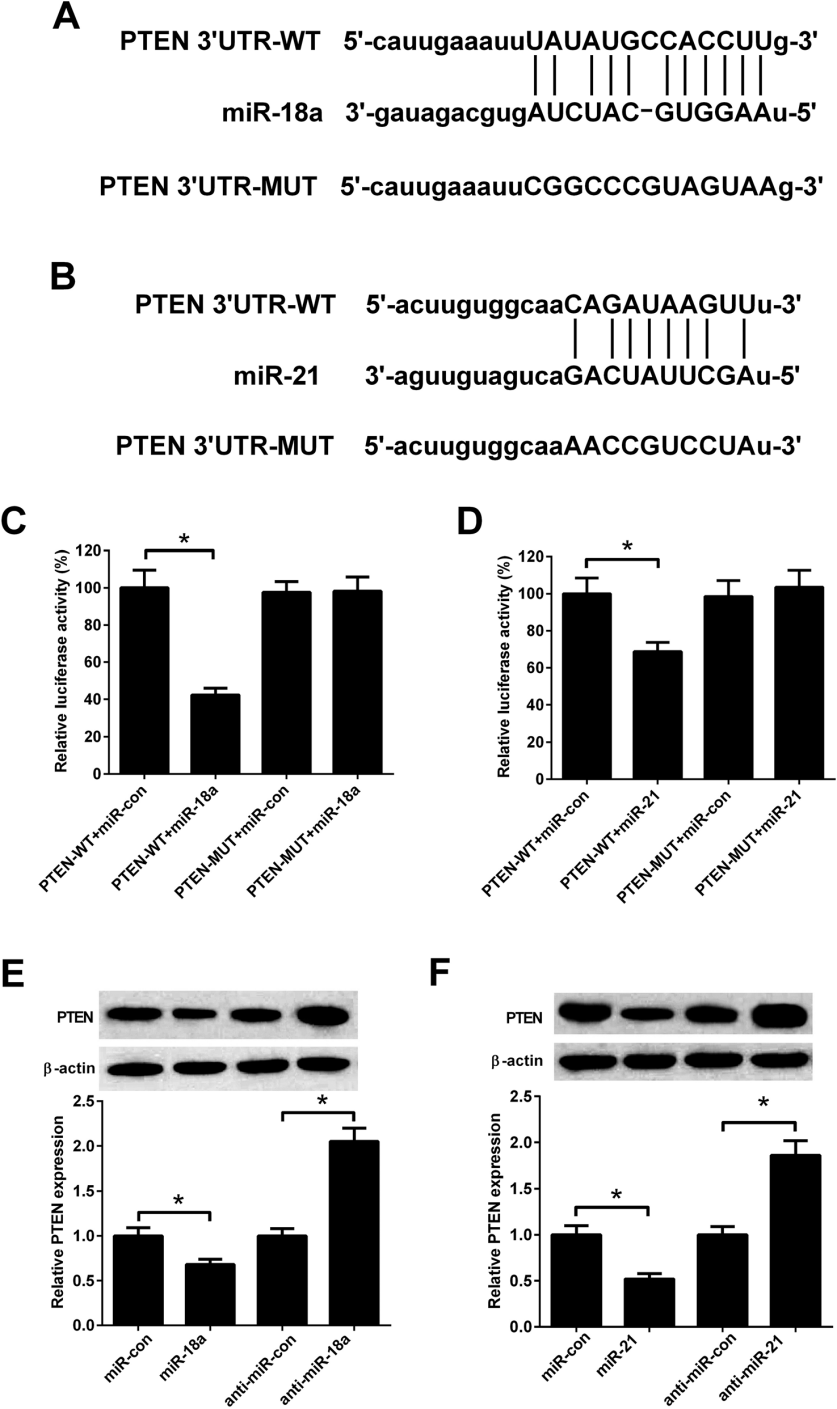
PTEN was a target of miR-18a and miR-21. (A) Binding sites between PTEN and miR-18a. (B) Binding sites between PTEN and miR-21. (C and D) Luciferase reporter assay was performed in A549 cells. (E and F) The expression levels of PTEN in A549 cells transfected with miR-18a inhibitor, miR-21 inhibitor, miR-18a mimics, or miR-21 mimics. *p < 0.05, *n* = 3.

Previous study reported that PTEN was a target of miR-18a and miR-21.^18,19^Fig. 6A and B showed predicted binding sites between miR-18a/miR-21 and PTEN. To evaluate whether PTEN was a direct target of miR-18a and miR-21 in NSCLC cells, A549 cells were co-transfected with PTEN-WT/PTEN-MUT and miR-18a/miR-21mimics. The luciferase activity was decreased in A549 cells co-transfected with PTEN-WT and miR-18a mimics compared with cells co-transfected with PTEN-WT and mimic control (Fig. 6C). Co-transfection with PTEN-WT and miR-21 mimics in A549 cells also decreased the luciferase activity compared with co-transfection with PTEN-WT and mimic control (Fig. 6D). The effect of miR-18a and miR-21 on the PTEN expression was detected by western blotting. As shown in Fig. 6E and F, miR-18a or miR-21 up-regulation decreased the PTEN expression, while miR-18a or miR-21 down-regulation increased the PTEN expression in A549 cells. The results indicated that PTEN was a direct target of miR-18a and miR-21 in A549 cells.

### CASC2 regulated the PTEN/PI3K/Akt pathway by down-regulating miR-18a and miR-21

**Fig. 7:**
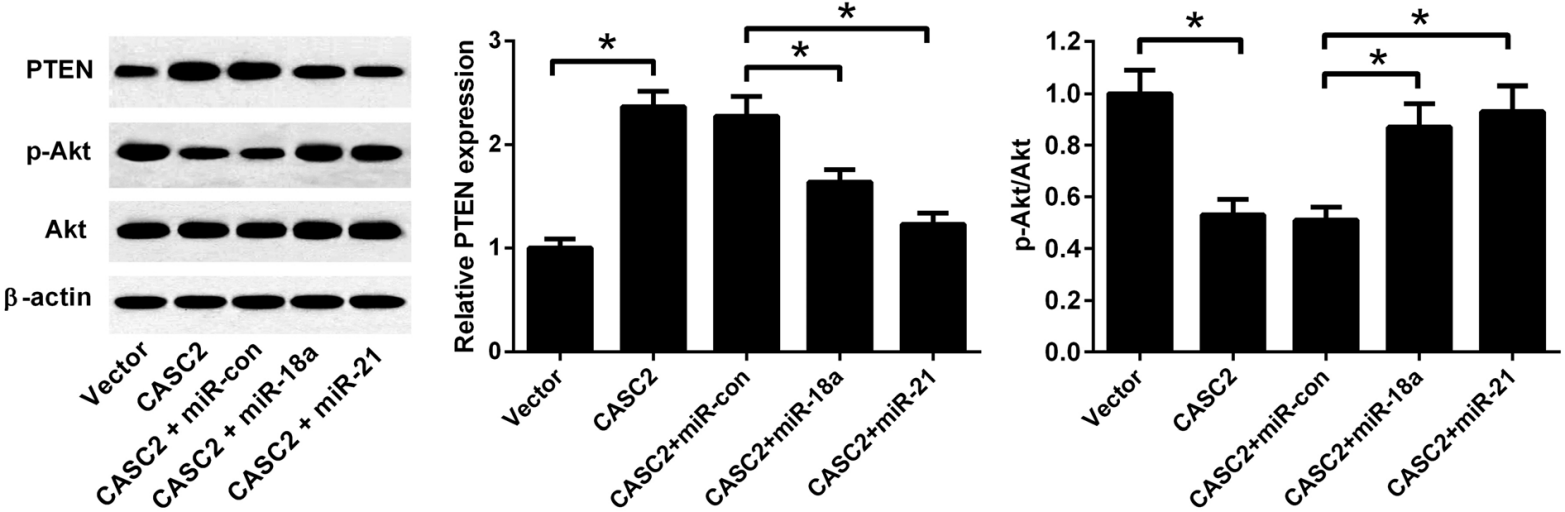
CASC2 regulated the PTEN/PI3K/Akt pathway by down-regulating miR-18a and miR-21. To investigate whether PTEN/PI3K/Akt pathway was involved in the effect of CASC2, A549 cells were co-transfected with pcDNA3.1-CASC2 and miR-18a/miR-21 mimics. The expression levels of PTEN, Akt, and p-Akt were detected by western blotting. *p < 0.05, *n* = 3.

To investigate whether PTEN/PI3K/Akt pathway was involved in the effect of CASC2, the expression levels of PTEN, Akt, and p-Akt were detected by western blotting. As shown in Fig. 7, CASC2 overexpression increased PTEN expression level and reduced the ratio of p-Akt/Akt. MiR-18a or miR-21 mimics attenuated the effect of CASC2 overexpression on the PTEN expression and ratio of p-Akt/Akt. The results indicated that CASC2 regulated the PTEN/PI3K/Akt pathway by down-regulating miR-18a and miR-21.

## Discussion

CASC2 is a well-known tumor suppressor.^7^ CASC2 expression was down-regulated in osteosarcoma tissue samples and cell lines and overexpression of CASC2 inhibited osteosarcoma cell proliferation, colony formation, and invasion ability.^20^ Zeng *et al.* suggested that the expression of CASC2 was reduced in the hepatocellular carcinoma (HCC) tissues and cell lines. CASC2 inhibited HCC cell viability, induced HCC cell apoptosis, and restrained the tumorigenesis of HCC cells.^21^ In the present study, we found that expression of CASC2 was decreased in NSCLC cell lines, compared with the 16-HBE cells. MiR-21 is a famous oncogene and has been found to be up-regulated in many cancers,^22,23^ including NSCLC.^14^ It has been reported that high plasma miR-18a is correlated with a worse disease-free survival and disease stage, indicating that miR-18a may serve as novel and promising prognostic biomarkers in patients with NSCLC.^24^ We also found that miR-18a and miR-21 were over-expressed in NSCLC cells.

A previous study showed that the expression of CASC2 was down-regulated in cervical cancer tissues and CASC2 expression was related to a shorter survival time and poorer clinicopathologic features. Overexpression of CACS2 inhibited cervical cancer cell proliferation and enhanced cisplatin-induced inhibition of cell proliferation. Besides, CACS2 was low-expressed in cisplatin-resistant cervical cancer tissues, compared to cisplatin-sensitive cancer tissues and CACS2 overexpression sensitized cisplatin-resistant cervical cancer cells to cisplatin.^25^ Another study suggested that CASC2 sensitized TMZ-resistant glioma cells to TMZ by up-regulating PTEN and down-regulating p-Akt protein through direct inhibiting miR-181a.^8^ CASC2 also reported to enhance TMZ cytotoxicity in glioma through autophagy inhibition by sponging miR-193a-5p and regulating mTOR expression.^9^ In our study, we found that low concentration of cisplatin (2.5 μM) was nontoxic to A549 and H1299 cells. CACS2 overexpression sensitized A549 and H1299 cells to cisplatin (2.5 μM). CASC2 overexpression enhanced the inhibition effect of cisplatin (2.5 μM) in SPC-A-1 cells. CASC2 overexpression also sensitized A549/CDDP cells to cisplatin (8 μM). Zhang *et al.* reported that miR-21 antisense oligonucleotide improved the sensitivity of human melanoma cells to cisplatin in an *in vitro* study.^26^ The results of another study indicated that overexpression of miR-21 promoted resistance to cisplatin in osteosarcoma cells, while suppression of miR-21 enhanced cisplatin cytotoxicity in osteosarcoma cells.^27^ Besides, miR-18a has an impact on chemotherapy resistance in esophageal cancer.^28^ In the present study, we found that down-regulation of miR-18a and miR-21 enhanced the inhibition effect of cisplatin on the viability of A549, H1299, and SPC-A-1 cells. Down-regulation of miR-18a and miR-21 also sensitized A549/CDDP cells to cisplatin (8 μM).

MiR-21 inhibitor suppressed retinoblastoma cell migration and invasion and regulated the expression of PTEN, PI3K, and p-Akt, suggesting that miR-21 inhibitor suppressed the progression of retinoblastoma *via* the PTEN/PI3K/Akt pathway.^29^ MiR-21 promoted cell proliferation, migration, and invasion and inhibited cell apoptosis of esophageal cancer cells through PTEN/PI3K/AKT pathway.^30^ Besides, miR-18a promoted proliferation of esophageal squamous cell carcinoma cells by increasing cylin D1 *via* regulating PTEN/PI3K/AKT/mTOR signaling axis.^31^ We found that PTEN was a direct target of miR-18a and miR-21 in NSCLC cells. CASC2 regulated the PTEN/PI3K/Akt pathway by down-regulating miR-18a and miR-21.

## Conclusion

In summary, we investigated the role of CASC2 in regulating cisplatin cytotoxicity in NSCLC cells. We found that CASC2 was low-expressed in NSCLC cell lines, and CASC2 enhanced the inhibition effect of cisplatin on cell viability. MiR-18a and miR-21 were the target of CASC2 in NSCLC. PTEN was found to be a direct target of miR-18a and miR-21 in NSCLC. The PTEN/PI3K/Akt pathway was involved in the effect of CASC2. The results indicated that CASC2 enhanced cisplatin-induced viability inhibition of NSCLC cells *via* the PTEN/PI3K/Akt pathway through suppressing of miR-18a and miR-21.

## Conflicts of interest

The authors declare that they have no conflict of interest.

## Supplementary Material
